# Pain Medication in Chronic Low Back Pain

**DOI:** 10.3390/life15050690

**Published:** 2025-04-23

**Authors:** Ali Jerjir, Frederik Nietvelt, Iris Smet, Nina D’hondt, Jean-Pierre Van Buyten

**Affiliations:** Multidisciplinary Pain Clinic, VITAZ Hospitals, 9100 Sint-Niklaas, Belgium; frederik.nietvelt@vitaz.be (F.N.); iris.smet@vitaz.be (I.S.); nina.dhondt@vitaz.be (N.D.); jean-pierre.vanbuyten@vitaz.be (J.-P.V.B.)

**Keywords:** chronic low back pain, CLBP, pharmacotherapy, polypharmacy, evidence-based management, clinical patient guidelines, CPG, opioid crisis, opioids

## Abstract

Chronic low back pain (CLBP) significantly impacts individuals’ quality of life and functional abilities. In non-oncological settings, CLBP is often treated for long periods using pharmacotherapy. This paper provides a comprehensive overview of pharmacological treatments for CLBP, detailing their mechanisms of action, adverse effects, and evidence supporting their use. We discuss various medication classes, including NSAIDs, acetaminophen, antidepressants, gabapentinoids, tramadol, major opioids, corticosteroids, antispasticity drugs, benzodiazepines, and antibiotics. Special emphasis is given to the opioid crisis, examining its history, the pathophysiology of opioid tolerance and dependence, the need for cautious opioid use, the key challenges in treatment and emerging medications for CLBP. We also share insights from our experiences with polypharmacy, commonly seen in CLBP patients, at a specialized pain centre in Belgium.

## 1. Introduction

Globally, the prevalence of all forms of low back pain is significant, with a point prevalence of approximately 11.9%, a one-month prevalence of about 23.2%, a one-year prevalence of around 38%, and a lifetime prevalence of approximately 39.9% [1]. In the United States of America (U.S.A.), the annual prevalence among adults is 10–30%, with a lifetime prevalence reported as high as 65–80%. Approximately 25% of U.S.A. adults experienced low back pain for at least 24 h in the past three months [2,3].

Chronic low back pain (CLBP) is a debilitating condition that affects a substantial portion of the adult population. It is defined as low back pain persisting for more than 12 weeks [2]. CLBP affects about 23% of the population globally, with 11–12% experiencing disability due to this condition [4]. The prevalence is highest in individuals in their third decade of life and continues to rise until the age of 60 to 65, i.e. during their active life span [3].

CLBP can be classified into non-degenerative CLBP, degenerative CLBP and CLBP of unknown origin, the last two formerly known as ‘non-specific’ CLBP. Non-degenerative CLBP is defined as pain caused by trauma, congenital spondylolysis, a tumor, an infection or an inflammatory process [1,2,3,4,5]. A substantial proportion of patients presenting with lower back pain in primary care settings are diagnosed with CLBP, highlighting its prevalence and significant impact on daily functioning and quality of life [2,5].

Low back pain significantly increases healthcare costs and causes productivity losses worldwide. In the U.S.A., direct healthcare costs attributable to low back pain were estimated at $26.3 billion in 1998, and the total economic cost, including indirect costs such as lost productivity, currently ranges from $560 to $635 billion annually [1,2,3]. Low back pain’s economic burden exceeds that of major conditions like heart disease, cancer, and diabetes [1]. Globally, it is the leading cause of disability and work absence according to the 2010 Global Burden of Disease study [2]. Although the number of physician visits for back pain has remained stable, the associated costs have increased significantly over the decades [4]. Among working adults in the U.S.A., the combined direct and indirect costs of low back pain amount to $6657 per patient annually [5]. This substantial economic impact highlights the need for improved management strategies to alleviate the burden of low back pain on both individuals and society.

Various factors are contributing to CLBP, with known influences of psychosocial, lifestyle, occupational, demographic, and genetic factors [1,2,3,4,5]. *Psychosocial factors* include anxiety, depression, fear-avoidance behaviour, and maladaptive coping strategies [1,2,3,4,5]. *Lifestyle factors* such as obesity, smoking, and sedentary behaviour are associated with increased risk [1,3,4,5]. *Occupational factors* include jobs involving prolonged sitting, heavy lifting, and exposure to vibration [1,2,3,5]. *Demographic influences* highlight the increased prevalence of CLBP with advancing age and lower educational levels [3,4]. *Genetic predispositions and comorbid medical conditions*, such as diabetes and rheumatoid arthritis, also contribute [1,4]. These factors, combined with stress and abnormal psychometric profiles, exacerbate and prolong CLBP.

The complexity of CLBP often necessitates a multifaceted treatment approach, incorporating both pharmacological and non-pharmacological strategies. Although weak evidence, if any, exists for the various treatment strategies, medication always has a key function. This review aims to outline the various classes of medications used in the management of CLBP, assessing their effectiveness and safety profiles. Emphasis is placed on the necessity for cautious use of opioids, given the significant public health issues arising from opioid dependence and abuse.

## 2. Materials and Methods

A literature search was conducted using PubMed to identify recent systematic reviews on clinical patient guidelines concerning medication use for CLBP. The search terms “clinical practice guidelines”, “chronic low back pain”, and “medication” were used. The inclusion criteria specified recent articles published in English within the last ten years, focusing on the pharmaceutical management of non-specific low back pain (i.e., CLBP due to degenerative spine disorders or of unknown origin) and providing recommendations for oral medications. CLBP was defined as lasting at least 12 weeks or 3 months. Initially, 77 articles were screened based on their abstracts, followed by a full-text review. We included a 2022 systematic review by Price et al. [6] and a 2018 systematic review by Oliveira et al. in the final analysis [7].

The review by Price et al. [6] analysed nine clinical practice guidelines (CPGs) on medication use for treating low back pain. To be included, the CPGs had to be published in English within the last five years and represent guidelines from various global sources. Excluded from the analysis were guidelines on structural LBP origins, injectable medications, and non-relevant or non-peer-reviewed studies. Additionally, two CPGs that focused solely on acute or subacute LBP were excluded from this chapter. The seven remaining guidelines that met our eligibility criteria are as follows:

- American College of Physicians (ACP) [8]
- Global Spine Care Initiative (GSCI) [9]
- Belgian Health Care Knowledge Centre (KCE) [10]
- North American Spine Society (NASS) [11]
- National Institute for Health and Care Excellence (NICE) [12,13]
- Toward Optimized Practice (TOP) [14]
- Veterans Affairs/Department of Defense (VA/DoD) [15]

In our analysis of the systematic review conducted by Oliveira et al. [7], we initially screened the nine clinical practice guidelines (CPGs) that addressed pharmacological therapy for CLBP. However, we excluded four guidelines due to their publication dates being over ten years old, two guidelines because they were not published in English, and one guideline that only briefly discussed the topic. Additionally, the two remaining guidelines that met the final criteria were also part of the systematic review by Price et al. [8,15].

Additionally, we searched for official recommendations of the World Health Organisation (WHO) on medication use for CLBP and came across the 2023 published guidelines for the non-surgical management of CPLBP in adults [16].

The full-text versions of the most recent version of these guidelines were analysed regarding specific recommendations. These were categorized as follows: “RECOMMENDED” when the guidelines recommend the use of a certain class of medication; “RECOMMEND AGAINST THE USE” if they advised against its use; and “NOT SPECIFIED” if the guidelines neither recommended nor opposed the use of the class or did not address it.

Medication categories were divided into NSAIDs, acetaminophen, antidepressants, gabapentinoids, tramadol, major opioids, corticosteroids, antispasticity drugs, benzodiazepines, and antibiotics.

## 3. Results

In the following section, we present an overview of the suggested mechanism of action, adverse effects and a summary of the CPG recommendations of the various classes of drugs commonly prescribed for the treatment of CLBP patients. A summary of CPG-based recommendations for different pharmacological classes used in CLBP is presented in Table 1, highlighting both consensus and discrepancies among guidelines.

### 3.1. Non-Steroidal Anti-Inflammatory Drugs (NSAIDs)

#### 3.1.1. Mechanism of Action

NSAIDs are diverse compounds with anti-inflammatory, analgesic, and antipyretic effects. They mainly work by inhibiting the COX enzyme, which is essential for producing prostaglandins and thromboxane [17]. COX-1 and COX-2 are two membrane-anchored isoenzymes. While COX-1 is constitutively expressed under physiologic conditions, COX-2 is induced under conditions of inflammation and injury [18].

#### 3.1.2. Adverse Effects

Gastrointestinal (GI) issues, cardiovascular risks, renal and hepatic concerns are associated with the use of NSAIDs. Adults over 45 years are advised to use gastric protection with proton-pump inhibitors [6]. COX-1 inhibition has an antiplatelet effect, while COX-2 inhibition provides anti-inflammatory, antipyretic, and analgesic benefits. However, COX-2 inhibition may lower gastrointestinal toxicity risk but increase cardiovascular side effects [19].

#### 3.1.3. Recommendations

- **ACP**: RECOMMENDED—1st line option—Moderate-quality evidence for small to moderate pain improvement and small functional improvement [8].
- **GSCI**: RECOMMENDED—1st line option—Small to moderate benefits and low harms [9].
- **KCE**: RECOMMENDED—Provides small clinical benefit; use lowest effective dose for the shortest period [10].
- **NASS**: RECOMMENDED—1st line option—Suggested for low back pain treatment [11].
- **NICE**: RECOMMENDED—Recommended with consideration for toxicity and patient risk factors, short term use [12,13].
- **TOP**: RECOMMENDED—2nd line option—Mild to moderately severe side effects; consider cardiovascular, renal, and gastrointestinal risks [14].
- **Va/DoD**: RECOMMENDED—Efficacy in pain relief for both acute and chronic LBP [15].
- **WHO**: RECOMMENDED—Conditional recommendation with moderate certainty evidence [16].

NSAIDs are widely recommended across various guidelines for the treatment of CLBP due to their small to moderate benefits in pain relief and function. They are often considered a first-line treatment option when nonpharmacologic treatments are inadequate. While generally effective, they come with potential risks such as gastrointestinal, liver, and cardiovascular issues. Therefore, it is advised to use the lowest effective dose for the shortest possible period [10,11,12,13,14,15,16].

### 3.2. Acetaminophen (Paracetamol)

#### 3.2.1. Mechanism of Action

Acetaminophen’s exact mechanism is unclear. It may inhibit central nervous system COX enzymes, particularly COX-2, but with low in vitro potency. COX-1 variant inhibition and enhancement of the descending serotonergic pathway are also suggested mechanisms. Its metabolites might also activate endocannabinoids and are also thought to affect ion channels in nerve cells that are involved in sensing pain and inflammation [20].

#### 3.2.2. Adverse Effects

Paracetamol is generally well-tolerated with minimal gastrointestinal side effects [6]. There is a potential for liver disease with long-term use [21].

#### 3.2.3. Recommendations

- **ACP**: RECOMMEND AGAINST THE USE—Low-quality evidence shows no difference compared to placebo [8].
- **GSCI**: RECOMMENDED—Small benefits and small harms; reasonable for short-term relief. 1st line option if contra-indications for NSAIDs [9].
- **KCE**: RECOMMEND AGAINST THE USE—Lack of significant benefit [10].
- **NASS**: NOT SPECIFIED—Insufficient evidence [11].
- **NICE**: RECOMMEND AGAINST THE USE—Lack of evidence of effectiveness [12,13].
- **TOP**: RECOMMENDED—1st line option—Negligible side effects but risk of liver toxicity with long-term use [14].
- **Va/DoD**: RECOMMEND AGAINST THE USE—No difference from placebo; potential liver damage [15].
- **WHO**: RECOMMEND AGAINST THE USE—No trials identified; potential harms outweigh benefits [16].

Acetaminophen is largely recommended against for the treatment of CLBP due to its lack of significant benefit compared to placebo. Most guidelines do not support its routine use, highlighting the potential for liver toxicity with long-term use [8,10,12,13,15,16]. However, it is noted as a reasonable option in the GSCI and TOP guidelines for short-term relief, particularly for patients who cannot take NSAIDs [9,14].

### 3.3. Antidepressants

#### 3.3.1. Mechanism of Action

**Tricyclic Antidepressants (TCAs):**

TCAs, such as amitriptyline and nortriptyline, increase the levels of norepinephrine and serotonin in the synaptic cleft, enhancing descending inhibitory pathways. They also block sodium channels, contributing to their analgesic effect [22].

**Serotonin-Norepinephrine Reuptake Inhibitors (SNRIs):**

SNRIs, such as duloxetine and venlafaxine, increase levels of both norepinephrine and serotonin, enhancing descending pain inhibition pathways [22]. Unlike SSRIs, duloxetine shows a rising dose–response curve [23].

#### 3.3.2. Adverse Effects

**Tricyclic Antidepressants (TCAs):**

**Common Side Effects:** TCAs cause anticholinergic effects such as urinary retention, constipation, dry mouth, drowsiness, blurred vision, tachycardia, memory disorders, and confusion in more than 60% of patients [22].

**Serotonin–Norepinephrine Reuptake Inhibitors (SNRIs):**

**Common Side Effects:** SNRIs can cause many side effects due to increased serotonin levels. Common issues include nausea, vomiting, constipation, somnolence, dry mouth, hyperhidrosis, anorexia, and fatigue. **Duloxetine specifically** often causes gastrointestinal disturbances [22].

#### 3.3.3. Recommendations

**Tricyclic Antidepressants**

- **ACP**: RECOMMEND AGAINST THE USE—Moderate-quality evidence shows no improvement in pain or function [8].
- **GSCI**: RECOMMENDED—Small to moderate benefits; suitable for patients with concomitant depression or anxiety [9].
- **KCE**: RECOMMEND AGAINST THE USE—Not recommended for routine use due to lack of strong evidence [10].
- **NASS**: RECOMMEND AGAINST THE USE—Not recommended for low back pain treatment [11].
- **NICE**: RECOMMEND AGAINST THE USE—No clinically important difference observed [12,13].
- **TOP**: RECOMMENDED—3rd line option—Small to moderate effect; side effects include drowsiness and anticholinergic effects [14].
- **Va/DoD**: RECOMMEND AGAINST THE USE—No significant benefit for pain or function [15].
- **WHO**: RECOMMEND AGAINST THE USE—Very low certainty evidence; potential for significant adverse effects [16].

**Serotonin–Norepinephrine Reuptake Inhibitors**

- **ACP**: RECOMMENDED—Moderate-quality evidence shows small improvements in pain and function [8].
- **GSCI**: RECOMMENDED—2nd line option—Small to moderate benefits and harms [9].
- **KCE**: RECOMMENDED—Some clinical benefit; recommendation not strong due to mixed evidence [10].
- **NASS**: RECOMMEND AGAINST THE USE—Not recommended for low back pain treatment [11].
- **NICE**: RECOMMEND AGAINST THE USE—No significant benefit; increased adverse events [12,13].
- **TOP**: NOT SPECIFIED—Inconclusive evidence [14].
- **Va/DoD**: RECOMMENDED—Moderate to high-quality evidence for pain and function improvement [15].
- **WHO**: RECOMMEND AGAINST THE USE—Large increased risk of adverse events; not favourable [16].

The use of TCAs for CLBP is generally not recommended. Clinical research suggests no significant improvement in pain or function, and their potential for adverse effects outweighs the benefits [8,10,11,12,13,15,16]. However, the GSCI guidelines consider TCAs a second-line option, and TOP as a third-line option, for patients with concomitant depression or anxiety, acknowledging small to moderate benefits [9,15].

Recommendations for SNRIs in CLBP management are mixed. While some guidelines recognize their small benefits in pain and function [8,9,10,15], others advise against their use due to a lack of significant benefit and an increased risk of adverse events [11,12,13,16]. SNRIs are occasionally considered for patients with additional depressive symptoms [9].

### 3.4. Gabapentinoids

#### 3.4.1. Mechanism of Action

Gabapentinoids, including gabapentin and pregabalin, are adjuvant analgesics and anti-epileptics. They bind to voltage-gated calcium channels, reducing calcium influx and neurotransmitter release. This action helps in neuropathic pain and inflammation. They also influence descending inhibitory pathways by reducing GABA transmission in the locus coeruleus [24].

#### 3.4.2. Adverse Effects

Gabapentinoids can cause fatigue, dry mouth, loss of balance, and concentration difficulties, and there is potential for abuse and dependence [6]. There is also an increased mortality directly linked to the intake of these drugs, especially in combination with opioids [25].

#### 3.4.3. Recommendations

- **ACP**: NOT SPECIFIED—Insufficient evidence [8].
- **GSCI**: NOT SPECIFIED—Uncertain evidence with associated CNS adverse events [9].
- **KCE**: RECOMMEND AGAINST THE USE—Lack of clinical benefit; increased risk of adverse events [10].
- **NASS**: NOT SPECIFIED—Insufficient evidence [11].
- **NICE**: RECOMMEND AGAINST THE USE—No improvement in symptoms; increased risk of adverse events [12,13].
- **TOP**: NOT SPECIFIED—Insufficient evidence [14].
- **Va/DoD**: RECOMMEND AGAINST THE USE—Mixed results; significant adverse effects [15].
- **WHO**: RECOMMEND AGAINST THE USE—Potential for significant adverse events; very low certainty evidence [16].

Gabapentinoids are generally recommended against for the treatment of CLBP [10,12,13,15,16]. The evidence for their efficacy is inconclusive, and they are associated with significant adverse effects.

### 3.5. Tramadol

#### 3.5.1. Mechanism of Action

Tramadol, a weak μ-opioid receptor analgesic, is a mix of (+)-tramadol and (−)-tramadol. The (+)-tramadol binds better to μ-opioid receptors and blocks serotonin reuptake, while the (−)-tramadol inhibits noradrenaline reuptake. It works by targeting μ-opioid, GABA, catecholamine, and serotonergic receptors [26].

#### 3.5.2. Adverse Effects

Tramadol can cause dizziness, drowsiness, gastrointestinal complaints, potential hypoglycaemia. Caution is needed when combined with tricyclic antidepressants (TCAs) or serotonin-norepinephrine reuptake inhibitors (SNRIs) [6].

#### 3.5.3. Recommendations

o **ACP**: RECOMMENDED—Moderate-quality evidence for moderate short-term pain relief, short term use [8].
o **GSCI**: NOT SPECIFIED—No specific recommendations provided [9].
o **KCE**: RECOMMEND AGAINST THE USE—Inconsistent results; increased risk of adverse events [10].
o **NASS**: RECOMMENDED—Use cautiously; limit to short duration [11].
o **NICE**: NOT SPECIFIED—Limited evidence [12,13].
o **TOP**: RECOMMENDED—4th line option—titrate slowly; risks include dizziness and gastrointestinal complaints [14].
o **Va/DoD**: NOT SPECIFIED—Insufficient evidence [15].
o **WHO**: NOT SPECIFIED—Equivocal balance between benefits and harms [16].

While tramadol shows moderate short-term pain relief, the inconsistency in results and potential for adverse effects limit its routine use. Some guidelines recommend tramadol but emphasize the need for cautious, limited-duration use [8,11,14].

### 3.6. Major Opioids

#### 3.6.1. Mechanism of Action

Opioids act on μ-, δ-, and κ-opioid receptors, triggering second messengers (e.g., cGMP, cAMP) and resulting in effects like analgesia and sedation. They are also substrates for P-glycoprotein at the blood-brain barrier, which affects their concentration at target sites [27].

#### 3.6.2. Adverse Effects

Opioids can cause constipation, dizziness, and fatigue, and have high rates of adverse events, including dependence, tolerance, and hyperalgesia [6].

#### 3.6.3. Recommendations

- **ACP**: RECOMMENDED—Small short-term improvement; use only if other treatments fail, limited to short durations [8].
- **GSCI**: RECOMMEND AGAINST THE USE—Risk of serious harms; not preferred [9].
- **KCE**: RECOMMEND AGAINST THE USE—High risk of adverse events; not recommended for routine use [10].
- **NASS**: RECOMMENDED—Cautiously limited to short durations [11].
- **NICE**: RECOMMEND AGAINST THE USE—Lack of long-term benefit; high risk of harm [12,13].
- **TOP**: RECOMMEND AGAINST THE USE—Not supported by evidence; consider only for severe chronic pain [14].
- **Va/DoD**: RECOMMEND AGAINST THE USE—Significant risks; no long-term benefit [15].
- **WHO**: RECOMMEND AGAINST THE USE—Moderate certainty evidence; potential harms outweigh benefits [16].

Major opioids are largely recommended against for CLBP due to their high risk of adverse events, including addiction and lack of long-term benefit [9,10,11,12,13,14,15,16]. They are considered only when other treatments have failed and should be used with caution and close monitoring [8,11]. Guidelines uniformly discourage their routine use, highlighting the importance of considering alternative therapies [8,9,10,11,12,13,14,15,16].

### 3.7. Systemic Corticosteroids

#### 3.7.1. Mechanism of Action

Corticosteroids, similar to cortisol, have strong anti-inflammatory and immunosuppressant effects. For low back pain, they are often injected into spinal structures, but they can also be administered orally, intravenously, or intramuscularly [28].

#### 3.7.2. Adverse Effects

Potential adverse effects include weight gain, osteoporosis, hypertension, diabetes, and increased risk of infections [6].

#### 3.7.3. Recommendations

- **ACP**: RECOMMEND AGAINST THE USE—Low-quality evidence showed no benefit [8].
- **GSCI**: RECOMMEND AGAINST THE USE—Lack of benefit; potential for moderate harms [9].
- **KCE**: NOT SPECIFIED—Not typically used; lack of sufficient evidence [10].
- **NASS**: RECOMMEND AGAINST THE USE—Not effective [11].
- **NICE**: RECOMMEND AGAINST THE USE—No improvement in pain or function; increased risk of adverse events [12,13].
- **TOP**: NOT SPECIFIED—Inconclusive evidence [14].
- **Va/DoD**: RECOMMEND AGAINST THE USE—No efficacy; potential harms [15].
- **WHO**: RECOMMEND AGAINST THE USE—Very low certainty evidence; potential harms [16].

Systemic corticosteroids are generally recommended against for CLBP [8,9,11,12,13,15,16]. There is no significant benefit in pain relief or functional improvement, and the potential for moderate to severe harms advises against their use [8,9,10,11,12,13,14,15,16]. Most guidelines do not support their routine use for CLBP.

### 3.8. Antispasticity Drugs

#### 3.8.1. Mechanism of Action

Baclofen activates GABAB receptors, reducing calcium influx and the release of excitatory neurotransmitters. Progabide acts on both classic and GABAB receptors. Glycine targets inhibitory receptors on spinal interneurons and motoneurons. Phenothiazines affect the brainstem to alter fusimotor fibre function. Phenytoin and carbamazepine reduce muscle spindle output, while dantrolene lowers calcium release from the sarcoplasmic reticulum, reducing muscle contraction [29].

#### 3.8.2. Adverse Effects

These drugs can cause drowsiness, dizziness, dry mouth, and fatigue, and have potential for dependency [6].

#### 3.8.3. Recommendations

- **ACP**: NOT SPECIFIED—Insufficient evidence [8].
- **GSCI**: NOT SPECIFIED—Uncertain evidence [9].
- **KCE**: RECOMMEND AGAINST THE USE—Conflicting evidence; significant adverse effects [10].
- **NASS**: NOT SPECIFIED—No specific recommendation [11].
- **NICE**: NOT SPECIFIED—Insufficient evidence [12,13].
- **TOP**: RECOMMENDED—May be appropriate for selected patients, limited to short durations; caution due to side effects [14].
- **Va/DoD**: RECOMMEND AGAINST THE USE—No evidence supporting long-term use [15].
- **WHO**: RECOMMEND AGAINST THE USE—Very low certainty evidence; potential adverse events [16].

The use of skeletal muscle relaxants in chronic low back pain is not strongly supported. There is insufficient evidence or conflicting data regarding their efficacy, and their potential adverse effects, particularly on the central nervous system, are a concern [8,9,10,11,12,13,15,16]. Only TOP guidelines recommend their short-term use for selected patients, with caution due to side effects [14].

### 3.9. Benzodiazepines

#### 3.9.1. Mechanism of Action

Benzodiazepines bind to specific receptors connected to GABA receptors on primary afferent terminals. This binding increases the GABA receptor’s affinity for the amino acid, enhances chloride ion flow across the membrane, and boosts presynaptic inhibition [29].

#### 3.9.2. Adverse Effects

Benzodiazepines can cause drowsiness, dizziness, fatigue, dependency, and withdrawal symptoms, and have potential for abuse [6].

#### 3.9.3. Recommendations

- **ACP**: NOT SPECIFIED—Low-quality evidence; high potential risks [8].
- **GSCI**: RECOMMEND AGAINST THE USE—Moderate harms; lack of evidence for benefit [9].
- **KCE**: RECOMMEND AGAINST THE USE—Risk of dependence; lack of evidence for benefit [10].
- **NASS**: NOT SPECIFIED—No specific recommendation [11].
- **NICE**: RECOMMEND AGAINST THE USE—Lack of evidence; potential for harm [12,13].
- **TOP**: NOT SPECIFIED—No specific recommendation [14].
- **Va/DoD**: RECOMMEND AGAINST THE USE—Insufficient evidence; significant risks [15].
- **WHO**: RECOMMEND AGAINST THE USE—No trials identified; potential harms [16].

Benzodiazepines are consistently recommended against for CLBP due to their lack of evidence for benefit and significant potential harms, including dependence [9,10,12,13,15,16]. Most guidelines do not support their use, reflecting concerns over their safety profile in CLBP management [8,9,10,11,12,13,14,15,16].

### 3.10. Antibiotics

#### 3.10.1. Mechanism of Action

The hypothesis for antibiotic use in CLBP suggests that a low-grade infection in the disc, caused by skin bacteria entering through a disc herniation, leads to infection, bone oedema (Modic type 1 changes), and severe pain [30].

#### 3.10.2. Adverse Effects

Antibiotics can cause gastrointestinal upset, allergic reactions, antibiotic resistance, and disruption of normal flora [6].

#### 3.10.3. Recommendations

- **ACP**: NOT SPECIFIED—Insufficient evidence [8].
- **GSCI**: NOT SPECIFIED—Insufficient evidence [9].
- **KCE**: RECOMMEND AGAINST THE USE—Lack of evidence; potential for adverse effects [10].
- **NASS**: NOT SPECIFIED—No specific recommendation [11].
- **NICE**: RECOMMEND AGAINST THE USE—Insufficient evidence; high potential for adverse events [12,13].
- **TOP**: RECOMMEND AGAINST THE USE—Not recommended for MRI Modic changes [14].
- **Va/DoD**: NOT SPECIFIED—No specific recommendation [15].
- **WHO**: NOT SPECIFIED—No trials identified [16].

Antibiotics are generally not recommended for the treatment of CLBP. The lack of supporting evidence and potential for adverse effects, including antibiotic resistance, advise against their use [8,9,10,11,12,13,14,15,16].

## 4. Discussion

### 4.1. Chronic Medication Management for CLBP Patients: Recommendations and Insights from a Leading Belgian Pain Centre

Patients with CLBP frequently rely on long-term medication regimens that pose sig-nificant risks of serious side effects over time. Despite the widespread use of these treat-ments, the evidence supporting their efficacy is often limited. This highlights the importance of further research to support a cautious, evidence-based approach to managing CLBP, prioritizing treatments with proven safety and efficacy profiles to mitigate long-term risks to patients.

When we see these patients at our pain centre for the first time, they are often on high doses of opioids, benzodiazepines, and anti-neuropathic drugs. This polypharmacy can blunt their emotions, making the tapering and discontinuation of these medications particularly challenging. As the cognitive haze from these drugs clears, unresolved emotional issues may surface, further complicating the withdrawal process. They often experience a mental fog that hinders active societal participation, leading to immobilization, social isolation, and financial difficulties due to work incapacity. Encouraging these patients to decrease their medication intake can be difficult, even when effective pain relief has been achieved.

Regarding current evidence on pharmacotherapy for treating CLBP, we recommend using a short-term course of NSAIDs. Tramadol may be used but is not preferred as an initial treatment; the dosage should be kept as low as reasonably achievable and for short-term use. SNRIs may be beneficial for patients with underlying mental health conditions, but the supporting evidence remains weak. We emphasize that pharmacotherapy for CLBP should not be viewed as a standalone treatment but rather as a tool to support a multimodal approach, incorporating physical therapy and cognitive behavioural therapy.

### 4.2. Challenges in the Treatment of CLBP

The search for a unique pain generator of CLBP is difficult as it can result from various factors, including mechanical, neuropathic, inflammatory, and psychological causes. Even when a clear nociceptive source is present, individuals with CLBP may be subjected to multiple other contributing factors. Therefore, phenotyping should consider functional dimensions beyond anatomical abnormalities. As mentioned above, given its often multifactorial causes, CLBP treatment should not rely solely on medication but should also incorporate a combination of cognitive behavioural therapy and physical therapy [31]. Yet this remains a challenge, as not all patients are open to a multimodal approach, and they frequently struggle to follow prescribed exercise routines due to factors like low motivation, limited time, and fear of worsening pain [32]. Furthermore, patients frequently experience long waiting times or a lack of access to essential services like physical therapy and pain management programs [33]. Moreover, scarce and weak evidence has been demonstrated for this multimodal approach.

The application of the WHO pain ladder, originally designed for cancer pain, to chronic non-cancer pain such as CLBP can be problematic due to the excessive use of opioids, leading to limited effectiveness and serious side effects. The pain ladder encourages a stepwise increase in pain medication, which can result in the prescription of opioids that may be unsuitable for long-term use in non-cancer-related pain. Studies confirm that opioids might provide short-term relief, but their long-term effectiveness is not proven, with even strong evidence for harm, especially at high doses. Severe side effects such as dependency, overdose, and increased pain sensitivity (hyperalgesia) can occur, along with other health complications like constipation, nausea, and reduced sexual function.

Due to the significant risks associated with long-term opioid use, alternative treatments and minimally invasive interventions should be prioritized. The current challenge with alternative therapies, such as platelet-rich plasma (PRP) or acupuncture, is their inconsistent effectiveness and lack of strong supporting evidence [34]. Early multidisciplinary assessment can improve patient outcomes and quality of life, reducing the risks of de-pendency and abuse. Comprehensive management of CLBP should aim to reduce pain, improve functionality, and ensure patient safety through judicious pharmacotherapy and multidisciplinary treatment integration. As there is no ‘one-size-fits-all’ solution, personalized treatment plans tailored to the underlying pain mechanisms and individual factors could be implemented.

### 4.3. Pathophysiology of Opioid Tolerance and Dependence

**Figure 1:**
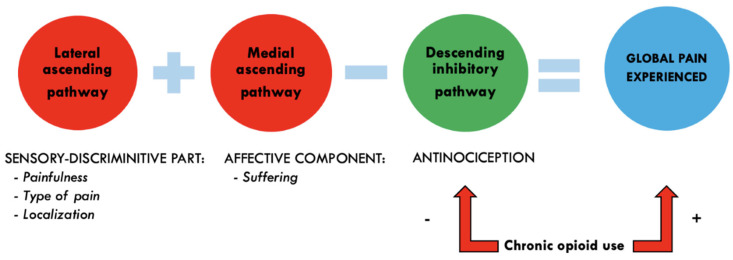
Global pain experienced is the sum of two ascending spinothalamic pathways minus the influence of the descending inhibitory pathway that must modulate these ascending pain pathways. Chronic opioid use underpins this inhibitory pathway, leading to an increase in pain experienced.

Pain perception involves two ascending spinothalamic pathways and a descending inhibitory pathway. The lateral ascending pathway is responsible for the sensory discriminative aspects of pain, such as its intensity and location. The medial ascending pathway processes the affective components, contributing to the suffering associated with pain [35]. The descending inhibitory pathway modulates the ascending pain signals, providing antinociception [36]. Effective pain inhibition relies on the proper functioning of all three pathways, as illustrated in Figure 1.

Chronic opioid therapy or abuse disrupts the descending pain modulatory system, a key component of pain control, by saturating receptors and inhibiting neurotransmitter release. This pathway includes the periaqueductal grey (PAG), rostral ventromedial medulla (RVM), and spinal cord dorsal horn. Normally, Mu-opioid receptor activation in these areas induces antinociception by exciting off-cells in the RVM, suppressing spinal cord pain. Mu-opioid receptors in the PAG are essential for developing opioid tolerance, affecting the entire descending pathway. Chronic opioid use leads to RVM adaptations, resulting in hyperalgesia [37,38].

Opioid dependence develops as patients transition from intermittent to continuous use. Initially, opioids provide analgesia and euphoria; however, over time, each dose is required to prevent hyperalgesia and dysphoria. This leads to tolerance and dependence, resulting in a state of persistent withdrawal. To avoid dysphoria and hyperalgesia, patients must increase their dosage, but eventually, no dose is adequate. This cycle of withdrawal symptoms drives cravings and opioid-seeking behaviours [39].

The complex interplay between opioid tolerance, dependence, and hyperalgesia underscores the necessity for careful management and the consideration of alternative pain relief strategies. Understanding these mechanisms is crucial for developing effective treatment plans that minimize the risks associated with chronic opioid use and enhance the overall quality of life for patients suffering from chronic low back pain.

### 4.4. Opioid Crisis

#### History

The opioid crisis now affects the entire Western world, not only the U.S.A. [40]. A significant catalyst for this crisis was a brief opinion paper by Porter and Jick in the New England Journal of Medicine in 1980, titled “Addiction rare in patients treated with narcotics” [41]. In response to growing concerns about adequate pain management, the WHO introduced the pain ladder in 1986, initially for cancer pain but gradually applied to chronic non-cancer pain (CNCP) as well [42]. In 1996, the American Pain Society’s campaign recognized pain as the 5th vital sign, further contributing to the issue [42].

The crisis intensified in North America with the aggressive promotion of the prescription of opioids, particularly the slow-release version of oxycodone, commercialized in 1995. This led to a 650-fold increase in sales between 1996 and 2010, which cannot be solely attributed to the “Pain as the 5th vital sign” campaign [42].

The production of synthetic fentanyl triggered a third wave of opioid overdose deaths. In Europe, oxycodone, fentanyl, and tramadol are the most prescribed opioids, with the ease of transdermal and transmucosal administration partly explaining the rise in fentanyl consumption [43].

### 4.5. Novel Medication in the Treatment of CLBP

#### 4.5.1. Cebranopadol

Cebranopadol is an investigational analgesic that acts as an agonist at both the μ-opioid and the nociceptin opioid peptide (NOP) receptors, providing a unique analgesic profile. NOP receptors play a crucial role in pain regulation, with their activation producing antinociceptive effects in the spinal cord. Clinical trials suggest cebranopadol provides effective dose-dependent pain relief in patients with moderate-to-severe CLBP [44]. Its side effects include nausea, dizziness, constipation, and headache. However, unlike traditional opioids, it is linked to fewer opioid-related side effects and may have a lower risk of abuse [45]. Currently, cebranopadol is not yet approved by the Food and Drug Administration (FDA) and remains under investigation. Ongoing studies continue to evaluate its full benefits and risks in chronic pain management.

#### 4.5.2. Tanezumab

Tanezumab is a monoclonal antibody designed to target nerve growth factor (NGF), a crucial regulator of pain signalling. By blocking NGF, it alleviates pain sensitivity without interacting with opioid receptors, offering a non-opioid alternative for chronic pain management. It has completed Phase III trials for CLBP, demonstrating moderate pain relief in patients with moderate-to-severe CLBP [46]. The primary safety concern is rapidly progressive osteoarthritis (RPOA), which may cause joint damage and necessitate joint replacement at the end. Due to this risk, the FDA has not approved tanezumab. Ongoing clinical trials are evaluating its risk–benefit profile, particularly in carefully selected patient populations.

#### 4.5.3. Suzetrigine

Suzetrigine is a new non-opioid analgesic that selectively inhibits the NaV1.8 sodium channel, a crucial component in pain signal transmission within the peripheral nervous system. By targeting this pathway, it alleviates pain without affecting opioid receptors, reducing the risk of addiction and other opioid-related side effects. Common side effects of suzetrigine include nausea, headache, dizziness, fatigue, and somnolence (drowsiness), though they are generally mild and tend to improve over time. Suzetrigine has recently been approved by the U.S. Food and Drug Administration (FDA) for the treatment of moderate to severe acute pain in adults. However, its use in chronic pain conditions, such as CLBP, is still under investigation, with ongoing studies evaluating its efficacy and safety profile in these settings.

## 5. Conclusions

The management of CLBP requires an evidence-based approach that minimizes reliance on long-term medication regimens. At this moment, such an approach is lacking. Therefore, emphasizing non-pharmacological treatments and carefully evaluating the risks and benefits of prescribed medications are essential steps to ensure patient safety and improve their overall quality of life.

Pharmacological treatments for CLBP vary in effectiveness and safety. For CLBP, NSAIDs are recommended for their pain relief and functional benefits but should be used at the lowest effective dose for the shortest duration due to potential risks. Tramadol can be used with caution for short-term relief. There is also some weak evidence that duloxetine may be considered, especially for patients with depression. Acetaminophen, TCAs, gabapentinoids, major opioids, systemic corticosteroids, antispasticity drugs, benzodiazepines, and antibiotics are generally not recommended due to a lack of significant benefits and potential adverse effects.

## Figures and Tables

**Table 1:** Summary of medication recommendations for the treatment of chronic low back pain across various clinical guidelines. Medication classes are listed on the vertical axis, and guidelines are listed on the horizontal axis with the following abbreviations: ACP (American College of Physicians), GSCI (Global Spine Care Initiative), KCE (Belgian Health Care Knowledge Centre), NASS (North American Spine Society), NICE (National Institute for Health and Care Excellence), TOP (Toward Optimized Practice), Va/DoD (Veterans Affairs/Department of Defense), and WHO (World Health Organization). The symbols indicate the following recommendations: + Recommended; +* Recommended for short use only; + (D) Recommended for patients with depression; - Recommend against use; / Not specified or no recommendation. Abbreviations: NSAIDs: Nonsteroidal Anti-Inflammatory Drugs; TCA: Tricyclic Antidepressants; SNRI: Serotonin-Norepinephrine Reuptake Inhibitors.

Medication Class	ACP	GSCI	KCE	NASS	NICE	TOP	Va/DoD	WHO
NSAIDs	+	+	+*	+	+*	+	+	/
Acetaminophen	-	+	+	/	-	/	-	/
TCA	-	+ (D)	-	-	-	/	/	/
SNRI	+	+ (D)	+	-	-	/	/	/
Gabapentinoids	/	/	-	-	-	/	-	/
Tramadol	+*	-	-	+*	-	+	+	/
Major Opioids	+*	-	-	+*	-	+	-	/
Corticosteroids	-	-	-	-	-	/	-	/
Antispasticity Drugs	/	/	/	/	+*	/	/	/
Benzodiazepines	/	/	-	-	-	/	-	/
Antibiotics	/	/	-	-	-	/	-	/

## Data Availability

No new data were created or analyzed in this study. Data sharing is not applicable to this article.
